# Harmonization of delirium severity instruments: a comparison of the DRS-R-98, MDAS, and CAM-S using item response theory

**DOI:** 10.1186/s12874-018-0552-4

**Published:** 2018-09-10

**Authors:** Alden L. Gross, Doug Tommet, Madeline D’Aquila, Eva Schmitt, Edward R. Marcantonio, Benjamin Helfand, Sharon K. Inouye, Richard N. Jones, for the BASIL Study Group, Sharon K. Inouye, Richard N. Jones, Tamara Fong, Tammy Hshieh, Edward R. Marcantonio, Annie Racine, Eva M. Schmitt, Dena Schulman-Green, Patricia A. Tabloski, Thomas Travison, Tatiana Abrantes, Brett Armstrong, Sylvia Bertrand, Angelee Butters, Madeline D’Aquila, Jacqueline Gallagher, Jennifer Kettell, Jacqueline Nee, Katelyn Parisi, Margaret Vella, Guoquan Xu, Lauren Weiner, Yun Gou, Douglas Tommet, Charles H. Brown, Sevdenur Cizginer, Diane Clark, Joseph H. Flaherty, Anne Gleason, Ann M. Kolanowski, Karen J. Neufeld, Margaret G. O’Connor, Margaret A. Pisani, Thomas Robinson, Joe Verghese, Heidi Wald, Sharon M. Gordon

**Affiliations:** 1 Department of Epidemiology, Johns Hopkins Bloomberg School of Public Health, 2024 E. Monument Street, Baltimore, MD 21205 USA; 2 Johns Hopkins University Center on Aging and Health, Baltimore, MD USA; 3 Departments of Psychiatry and Human Behavior and Neurology, Warren Alpert Medical School, Brown University, Providence, RI USA; 4 Aging Brain Center, Institute for Aging Research, Hebrew SeniorLife, Boston, MA USA; 5 Department of Medicine, Beth Israel Deaconess Medical Center, Harvard Medical School, Boston, MA USA; 6 Department of Emergency Medicine, University of Massachusetts Medical School, Worcester, MA USA

**Keywords:** Delirium, Severity, Elderly, Psychometrics, Item response theory

## Abstract

**Background:**

This study aimed to describe the level of agreement of three commonly used delirium instruments: the Delirium Rating Scale-Revised-98 (DRS-R-98), Memorial Delirium Assessment Scale (MDAS), and Confusion Assessment Method-Severity (CAM-S).

**Methods:**

We used data from a prospective clinical research study, in which a team of trained lay interviewers administered each instrument along with supporting interview and cognitive assessments in the same group of patients daily while in the hospital (*N* = 352). We used item response theory methods to co-calibrate the instruments.

**Results:**

The latent traits underlying the three measures, capturing the severity of a delirium assessment, had a high degree of correlation (r’s > .82). Unidimensional factor models fit well, facilitating co-calibration of the instruments. Across instruments, the less intense symptoms were generally items reflecting cognitive impairment. Although the intensity of delirium severity for most in the sample was relatively low, many of the item thresholds for the delirium severity scales are high (i.e., in the more severe range of the latent ability distribution). This indicates that even people with severe delirium may have a low probability of endorsing the highest severity categories for many items. Co-calibration enabled us to derive crosswalks to map delirium severity scores among the delirium instruments.

**Conclusion:**

These delirium instruments measure the same underlying construct of delirium severity. Relative locations of items may inform design of refined measurement instruments. Mapping of overall delirium severity scores across the delirium severity instruments enabled us to derive crosswalks, which allow scores to be translated across instruments, facilitating comparison and combination of delirium studies for integrative analysis.

**Electronic supplementary material:**

The online version of this article (10.1186/s12874-018-0552-4) contains supplementary material, which is available to authorized users.

## Background

Delirium -- the acute onset of inattention, global cognitive impairment, and frequent co-occurring psychomotor, behavior or perceptual disturbance -- is a common, serious, and often preventable complication among hospitalized and institutionalized older adults [1]. An estimated 12 million older Americans (> 65 years-old) experience an episode of delirium each year [2, 3]. Delirium is distressing to patients and their families [4], prolongs hospital stays, delays rehabilitation, and is associated with excess risk of dementia and death [3, 5].

In proportion to its personal and public health impact, delirium is an understudied neuropsychiatric disorder [2, 3]. While delirium is preventable [6], effective treatments remain elusive [2, 3]. Published treatment trials have been hampered by discrepant findings and important methodologic limitations [7, 8]. One important methodological distinction is that for delirium prevention trials, delirium incidence is an appropriate primary outcome, while for delirium treatment trials, outcomes that track the course of delirium over time are essential. A set of responsive severity outcome measures that are valid reflections of symptom severity would greatly facilitate delirium treatment trials. Finding measures that correlate with underlying pathophysiologic mechanisms would also help advance our fundamental understanding of delirium and to develop more pathophysiologically based treatments. Both strategies would require the availability of sophisticated and fine-grained delirium severity measures.

Delirium severity ratings have important clinical and research applications because they provide directly graded, continuous measures, are correlated with clinical outcomes, and can be powerful prognostic predictors. Clinical uses of such measures include tracking change in delirium and its symptoms over time, identifying the earliest onset of clinically significant delirium, monitoring recovery from an episode, gauging patient and caregiver needs, and maintaining safe staffing levels in hospitals or at home. Additionally, there are important research implications for delirium severity measures. Higher severity leads to increased risk of long-term cognitive decline; thus, serial monitoring of severity is important in studies. Such measures provide key outcome measures for clinical trials and prognostic studies. These types of measures could also demonstrate graded impact of severity on health care delivery and costs. Additionally, correlations of delirium severity measures with biomarkers may help advance pathophysiologic understanding of delirium.

A multitude of measures have been designed to measure the severity of delirium. Currently used delirium severity measures provide mixed coverage of delirium signs and symptoms. In our review of the literature (Jones et al. in preparation), we find that the most frequently cited multiple item summative instruments used to rate the severity of a delirium episode include the Confusion Assessment Method (CAM and CAM-S [9, 10]), the Delirium Rating Scale (DRS, and DRS-R-98 [11, 12]), and the Memorial Delirium Assessment Scale (MDAS [13]). The goal of this paper is to describe the measurement properties and correspondence of these three most commonly used delirium severity instruments. We do this in the context of a clinical research study of older hospitalized patients, where a team of trained lay interviewers administered each instrument along with supporting interview and cognitive assessments to the same group of patients daily while in the hospital. We make use of modern psychometric methods to describe the extent to which these instruments measure the same underlying attribute.

## Methods

### Aim and design

The goal of this project was to describe the relationship of three delirium severity instruments to one another. We accomplished this within a prospective observational study of hospitalized older adults using modern psychometric methods including factor analysis and item response theory.

### Study sample and procedure

The Better Assessment of Illness (BASIL) study is an ongoing prospective cohort study, with planned one-year follow-up of all study participants. Eligible BASIL participants were aged 70 years or older, English speaking, and residing within 40 miles of and admitted or transferred to the medical or surgical services and Beth Israel Deaconess Medical Center (BIDMC), Boston, MA. Both emergency and elective admissions were enrolled. Exclusion criteria included evidence of active alcohol abuse, diagnosis of schizophrenia or other psychotic disorder, severe deafness, nonverbal condition, immediate discharge plans, or terminal condition. Of patients enrolled between October 2015 and March 2017, *N* = 352 patients had some data on each of the three delirium severity instruments for at least one day and comprise the sample for the present study. Trained lay interviewers assessed participants for delirium daily while hospitalized, and each participant provided between 1 and 15 daily assessments. Interviewers used a structured protocol to assess delirium and underwent in-depth training before administering the cognitive tests and coding the three delirium severity instruments. Inter-rater reliability checks were conducted regularly during the study to assure high quality ratings on these measures.

### Conceptualization of the measurement of delirium severity and intensity

We distinguish the intensity and severity in the context of delirium. As used in the field, delirium severity is a broad concept that encompasses the intensity of multiple symptoms. To allow for the conceptual distinction of the varied aspects of delirium severity, we use the term intensity to describe the distribution of cognitive, behavioral, psychiatric, and functional signs and symptoms associated with a single assessment. Intensity is viewed as existing along a continuum, ranging from the absence of signs and symptoms of delirium to highly disruptive behaviors or severely impairing symptoms. Intensity occupies the same conceptual space as the sum of symptom ratings represented by the total scores on the DRS-R-98, MDAS, and CAM-S. However, each instrument has its own metric that is defined by the particular selection of the number of domains assessed and the specific definitions used to characterize different rating levels of each sign or symptom. The result is that the summary scores may not necessarily have a direct relationship to one another, and thus, it can be challenging to integrate results from different studies using different measurement instruments.

We address this challenge by using item response theory methods to define the metric of underlying intensity of the individual delirium signs and symptoms. We use a common item equating design [14] and item response theory methods [15] to define an underlying delirium intensity metric that is equal across all three instruments. Moreover -- and as a consequence of using item response theory to define the metric for intensity -- the intensity metric can be used to describe the signs or symptoms; that is, the metric can be used to describe the level of impairment expressed by an individual patient at a discrete time of observation. Also, the metric can be used to describe specific items that assess the signs or symptoms of delirium: namely the region of the intensity metric that is measured symptoms are rated in lower or higher categories. In this way, the conceptualization of delirium severity represents a composite of the intensity of individual symptoms.

### Delirium severity assessment instruments

The DRS-R-98, MDAS, and CAM-S were used to rate delirium severity following brief interviews. The instruments encompass some overlap in features of delirium yet are distinctive. The instruments vary in their coverage of delirium severity domains and the manner in which the intensity of a specific symptom is rated. Thus, the interview source items used to score the three instruments vary.

The DRS-R-98 [16] uses family, chart, and nurses to identify and rate the severity of delirium according to 13 items: sleep/wake cycle disturbance, perceptual disturbances and hallucinations, delusions, lability of affect, language, thought process abnormalities, motor agitation, motor retardation, orientation, attention, short-term memory, long-term memory, and visuospatial ability The ratings for each item include 0 (no impairment), 1 (mild), 2 (moderate), and 3 (severe impairment). An overall score is created by summing the score for each item and higher scores indicate greater severity of delirium.

The MDAS [13] assesses the severity of delirium using 10 items on a four point scale (0 to 3) similar to the DRS-R-98 scale described above. The items assess diagnostic criteria of delirium according to the Diagnostic and Statistical Manual of Mental Disorders, Fourth Edition (DSM-IV [17]). MDAS items include reduced level of consciousness, disorientation, short-term memory, impaired digit span, reduced ability to maintain and shift attention, disorganized thinking, perceptual disturbance, delusions, psychomotor activity, and sleep/wake cycle disturbances. Again, an overall score is created by summing each item score; scores of 13 or higher indicates delirium, and higher scores indicate greater delirium severity.

The CAM [9] consists of 10 operationalized items from the DSM-III: acute onset and fluctuation, inattention, disorganized thinking, altered level of consciousness, disorientation, memory impairment, perceptual disturbances, psychomotor agitation or retardation, and altered sleep-wake cycle. The CAM diagnostic algorithm of delirium requires the presence of acute onset and fluctuation and inattention, and either disorganized thinking or altered level of consciousness. The severity scores derived from the CAM include the CAM-S [10] long form (10 items from the full CAM) and short form (four items from CAM diagnostic algorithm). Scoring systems allow for the quantification of the severity of delirium. The CAM-S is scored by rating CAM features, except acute onset or fluctuation, on a three-point scale: 0 (absent), 1 (mild), or 2 (marked). Acute onset or fluctuation is rated 0 (absent) or 1 (present). The composite (sum) short form scores range from 0 to 7, and the long form scores range from 0 to 19, with higher scores indicating greater delirium severity.

### Delirium rating

The daily hospital interviews in the BASIL study included a variety of formal questions and cognitive tests. These interview items were administered by the same raters, in same ordering, for every participant. In addition, interviewers gathered observational evidence throughout the hospital visits. Inter-rater reliability was assured through initial training and ongoing standardization approaches of the raters throughout the study. We modified the DRS-R-98 to accommodate assessment by trained lay interviews, rather than clinicians as originally intended by its developers. The scoring of the DRS-R-98, MDAS, and CAM-S was informed by source items (e.g., interview questions or cognitive tests) and informal observations. In addition to questioning and cognitive testing, the rated items in the three instruments include symptoms rated based on observations of the patient noted throughout the interview. Interviewers were trained to take extensive observational notes during the entire course of the patient visit. Some rated items, such as abnormal level of consciousness and psychomotor agitation/retardation, were not associated with specific formal source items and were scored using observational evidence only. The rated items of the DRS-R-98, MDAS, and CAM-S have a differing number of response categories. Rated items from the DRS-R-98 and MDAS have four response categories (*not present*, *mild*, *moderate*, and *severe*), while the rated items in the CAM have three response categories (*not present*, *mild*, *marked*). To facilitate co-calibration across delirium severity instruments, for initial modeling steps, described in the analysis plan, the response categories were dichotomized to not present vs any symptom, except for sleep disturbance. Because most participants endorsed mild sleep disturbance, we dichotomized it as not present or mild vs moderate or severe to identify only the more severe cases of sleep disturbance. The original polytomous items were used during the final scoring procedure. Items that were coded as *uncertain*, *refused* or *don’t know* were set to missing.

### Harmonization approach

Harmonization is a broad term that describes a process of addressing differences in measurement or assessment that could involve procedural, rational, or statistical approaches [18]. Our approach was to use statistical methods for harmonization using item response theory (IRT) methods [14]. IRT is a latent variable technique that describes a large family of statistical approaches that are used to describe the relationship between item responses (in our case, delirium sign or symptom ratings) and the latent trait presumed to underlie those responses (in our case, delirium intensity). We *equate* the latent trait distributions underlying each of three delirium assessment instruments, and report *links* (or crosswalks) among the total scores derived from each instrument. We use a blended common person and common item design [14, 19].

**Fig. 1:**
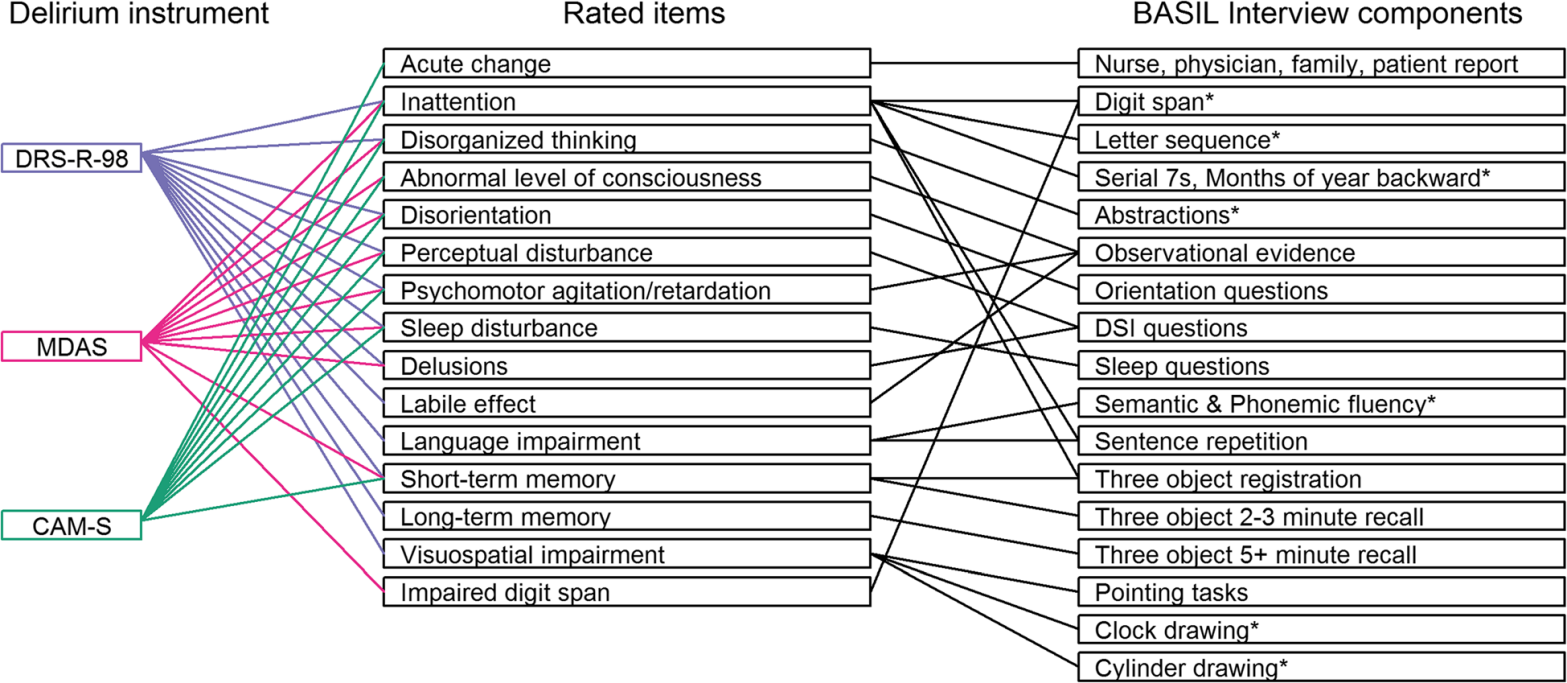
Map of instrument items, interview and rater assessments. In addition to formal interview questions, observational evidence was used to rate all domains. Orientation was assessed by questions regarding orientation to time and place. Sleep was assessed with a series of 6 questions about sleep quality in the last 24 h. The DSI (Delirium Symptom Interview) asked seven questions about distorted perceptions over the past 24 h. * Asterisked cognitive items are from the Montreal Cognitive Assessment (MoCA [23])

Seven items were shared among the three instruments, while the other items were shared among two instruments or not shared at all. Shared means that the content of the symptom was similar across instruments. The shared and unshared delirium items were associated with varying source material, as outlined in Fig. 1. Shared items among the three instruments included attention, disorganized thinking/thought process abnormality, orientation, perceptual disturbance, psychomotor agitation/retardation, sleep/wake cycle disturbance, and memory impairment. The assessment of attention involved digit span repetition (forward and backward), sentence repetition, and days of the week and months of the year backward. Source items used to score orientation assessed orientation to year, season, month, day of the week, date, city or town, name of place, and type of place. Sleep/wake cycle disturbance was assessed using six questions related to sleep cycle in the past 24 h. A three-object recall was used to evaluate memory impairment.

### Analysis plan

To perform statistical harmonization of the instruments, we used co-calibration based on IRT in four modeling steps.

First, a unidimensional confirmatory factor analysis (CFA) model was fit to each of the three instruments separately using dichotomized versions of the set of seven shared items among the delirium instruments. We used Mplus software (version 8, Muthén & Muthén, Los Angeles CA) and a robust maximum likelihood estimator and logit link for dichotomous items, and thereby implement a two parameter logistic IRT model with this CFA [20]. The goal of this step is to assess the appropriateness of a unidimensional model.

In the second modeling step, another unidimensional CFA model was fit separately to each of the three instruments using dichotomized versions of all the rated items of the instrument, with the factor loading and location parameters of the shared items constrained to the estimated values from the first model. The goal of this step is to determine item parameters for the non-shared items while constraining the measurement model to conform to the unidimensional model estimated using the shared items.

In a third modeling step, the items in the MDAS and the CAM-S were linked to the DRS-R-98 using the Haebara IRT linking procedure [21]. The shared items used to equate the MDAS to the DRS-R-98 were: disorganized thinking, orientation, perceptual disturbance, sleep/wake cycle disturbance, delusions, and memory impairment. The shared items used to equate the CAM-S to the DRS-R-98 were: attention, disorganized thinking, orientation, perceptual disturbance, psychomotor agitation, sleep/wake cycle disturbance, and psychomotor retardation. Using factor loading and location parameters from the set of common rated items in the earlier steps causes the resulting factor scores for delirium severity to be on the same metric for each instrument. This IRT linking procedure places the item parameters on the same metric across the three instruments, using the shared items as anchors.

In a fourth modeling step, after the dichotomous version of the items in the MDAS and CAM-S were equated to the DRS-R-98 in the above modeling steps, a unidimensional CFA model for each delirium instrument was fit to the polytomous version of the items. The factor loading and the first threshold for each item were constrained to the equated item parameters from the third step, and the remaining thresholds were freely estimated. This step allows us to obtain estimates for the higher category thresholds assuming the measurement slopes and first thresholds are valid population parameters. The two-stage approach (first estimate slopes and thresholds for dichotomized items, then given these estimate thresholds for higher categories) was necessitated to stabilize the estimates in the presence of a small number of responses in the higher categories.

Once delirium severity was linked across the DRS-R-98, MDAS, and CAM-S (long form and short form), we described item characteristics for each rated item, the agreement between severity scores, test characteristic curves, and the test information or precision of each measurement model. These methods illustrate the relationship between characteristics of the items and severity scores, thereby informing the complex construct of delirium severity as measured by the present instruments.

As commonly practiced, the mean of the latent delirium severity factor from each model was set to 0 with variance 1. The CFA models used a logit link and a maximum likelihood estimator. Models were also run using the weighted least squares (WLSMV) estimator to obtain Root Mean Square Error (RMSEA) and Comparative Fit Index (CFI) fit statistics [22]. RMSEAs below 0.05 and CFIs above 0.95 indicate excellent model fit. Since models used repeated daily delirium assessments, in factor analyses we used robust estimation procedures in Mplus to address the non-independence of observations resulting from clustering on person in the repeat assessments.

## Results

Study participants were on average 80 years old and had more than a high school education (mean 14.5 years of education) (Table 1). The average length of a hospital stay was 9 days (mean 8.6 +/− 6.4 days). The *N* = 352 participants contributed a total of 1178 daily observations to the present study. Most participants were female (59%), non-Hispanic white (85%), and currently unmarried (60%) but not living alone (62%); 29% had dementia.

**Table 1:** Baseline characteristics of the BASIL sample (*N* = 352)

Characteristic	Mean (SD) or *N* (%)
Age, years, mean (SD)	80.3 (6.8)
Female sex, *n* (%)	203 (57.7)
Non-white race, *n* (%)	52 (14.8)
Years of education, mean (SD)	14.5 (3.0)
Married, *n* (%)	139 (39.7)
Lives alone, *n* (%)	135 (38.6)
Lives in nursing home, *n* (%)	13 (3.7)
Dementia, *n* (%)	101 (28.7)
Charlson comorbidity score, mean (SD)	2.2 (2.2)
Surgical patient, *n* (%)	102 (29.0)
CAM delirium (ever), *n* (%)	68 (19.3)

Descriptive statistics for the DRS-R-98, MDAS, and CAM-S are presented in Table 2. We first evaluated evidence for unidimensionality of indicators for each instrument. Model fit was excellent in CFA models using common items among the instruments (CFI ≥ 0.97; RMSEA≤0.04) (Table 3). Fit of the CFA models using all rated items were somewhat lower except for the MDAS but continued to demonstrate a moderate level of fit (CFI ≥ 0.92; RMSEA≤0.09).

**Table 2:** Descriptive statistics of the DRS-R-98, MDAS, and CAM-S from all hospital interviews: Results from the BASIL study (*N* = 1178 daily observations)

Delirium instrument	Mean	SD	Minimum	Median	Maximum
DRS-R-98	4.7	4.3	0	3	28
MDAS	3.9	3.3	0	3	22
CAMS - Long form	2.3	2.4	0	1	14
CAMS - Short form	0.7	1.3	0	0	6

Note: *SD* Standard deviation

**Table 3:** Model fit statistics for unidimensional models using different item sets: Results from the BASIL study (*N* = 1178 daily observations)

Indicator inclusion:	Shared Items		All Items	
Delirium Instrument	CFI	RMSEA	CFI	RMSEA
DRS-R-98	0.98	0.03	0.97	0.08
MDAS	0.97	0.04	0.98	0.02
CAM-S	0.99	0.02	0.90	0.10

*CFI* Comparative Fit Index, *RMSEA* Root Mean Square Error of Approximation. Rated items that were common and unique to each instrument are shown in Fig. 1. Good model fit is typically defined as CFI values greater than 0.95 and RMSEA values less than 0.05. Poor fit for all-item models signal inadequacy of the unidimensionality assumption

**Fig. 2:**
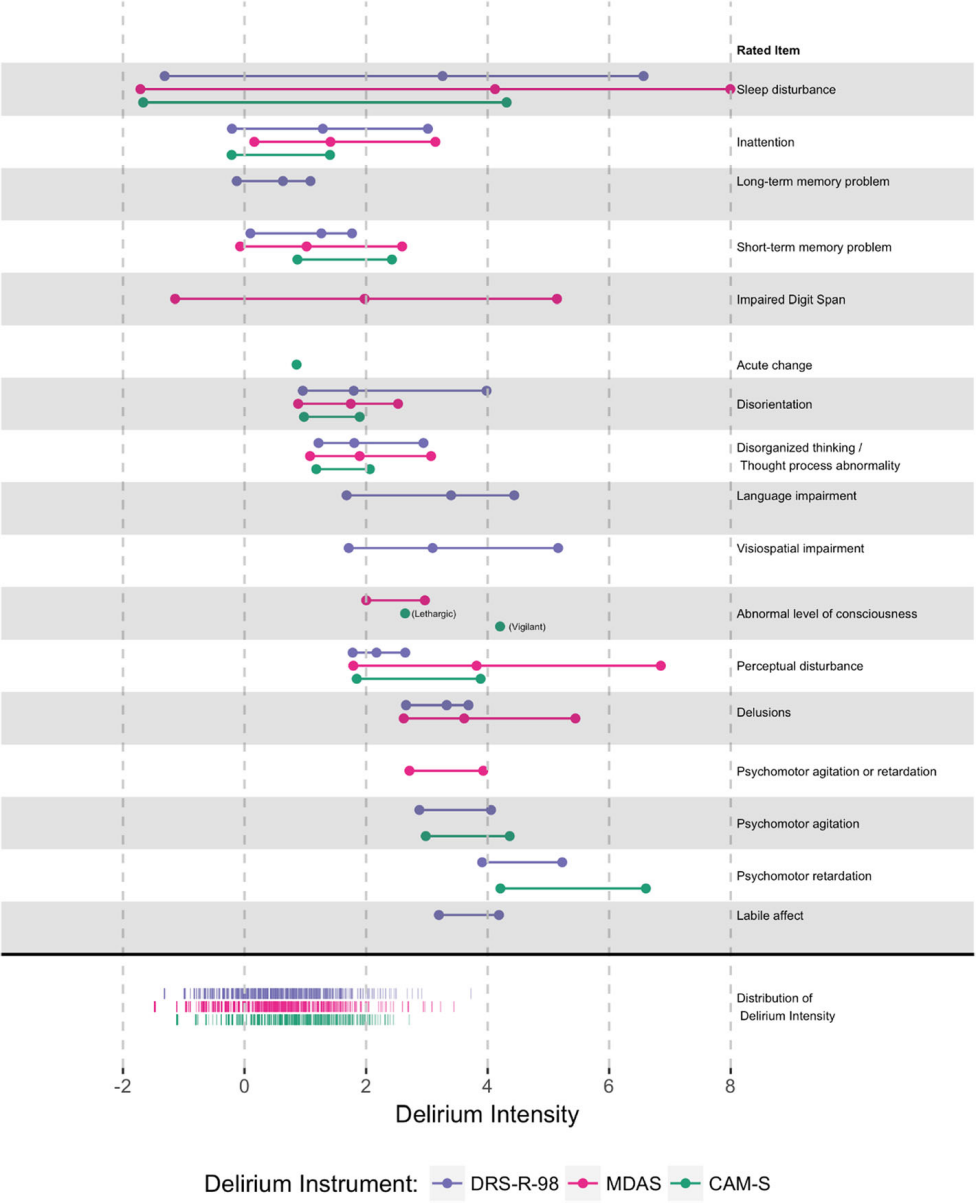
Item-person map: Results from the BASIL study (*N* = 1178 daily observations). Model-estimated item location parameters for each delirium instrument, grouped by rated item, are plotted along the range of delirium intensity. The distribution of delirium intensity scores in the sample is denoted on the bottom of the figure by the purple (DRS-R-98), pink (MDAS), and green (CAM-S) frequency distributions

Figure 2 presents model-estimated item location parameters for each instrument, grouped by the rated item, along the range of delirium intensity. The distribution of delirium intensity factor scores in the sample is denoted on the bottom of the figure by purple (DRS-R-98), pink (MDAS), and green (CAM-S). The item location parameters map to the level of delirium intensity. More intense items fall to the right, while less intense items fall to the left. Using a threshold of 4 SD units above the mean, the more intense categories of sleep disturbance, impaired digit span, language impairment, visuospatial impairment, perceptual disturbance, delusions, and psychomotor agitation/retardation are more likely to be seen in more severe cases of delirium.

Figure 2 demonstrates three important points. First, across instruments sleep disturbance and cognition relate to relatively less severe delirium. Second, despite the relatively low delirium intensity in most of the sample, highlighted by the colored frequency distributions of scores at bottom ranging between − 1 and 2 standard deviation units from the sample mean, most of the item thresholds provide information in the higher range of the spectrum. This indicates that even people with severe delirium have a low probability of endorsing many items. Rated items with minimum thresholds more intense than delirium intensity for most of the sample include abnormal level of consciousness, perceptual disturbance, delusions, psychomotor agitation/retardation, and labile affect. In other words, most of the patients in this sample did not experience these symptoms even if they were diagnosed with delirium. Third, Fig. 2 shows that differences in scoring of the instruments are possible at similar levels of delirium intensity. For example, thresholds are different for perceptual disturbance across delirium instruments. Although small numbers of participants at the most extreme levels of items account for the higher thresholds, this difference across instruments is partly attributable to differences in the exact wording of the questions or coding of answering choices in each of the respective instruments. For example, CAM-S ratings are more often based on an overall determination about whether the delirium symptom prolonged or interfered with the interview. By contrast, the DRS-R-98 and MDAS provide specific scoring instructions to rate each item, which differ substantially in scoring, i.e., perceptual disturbances.

**Fig. 3:**
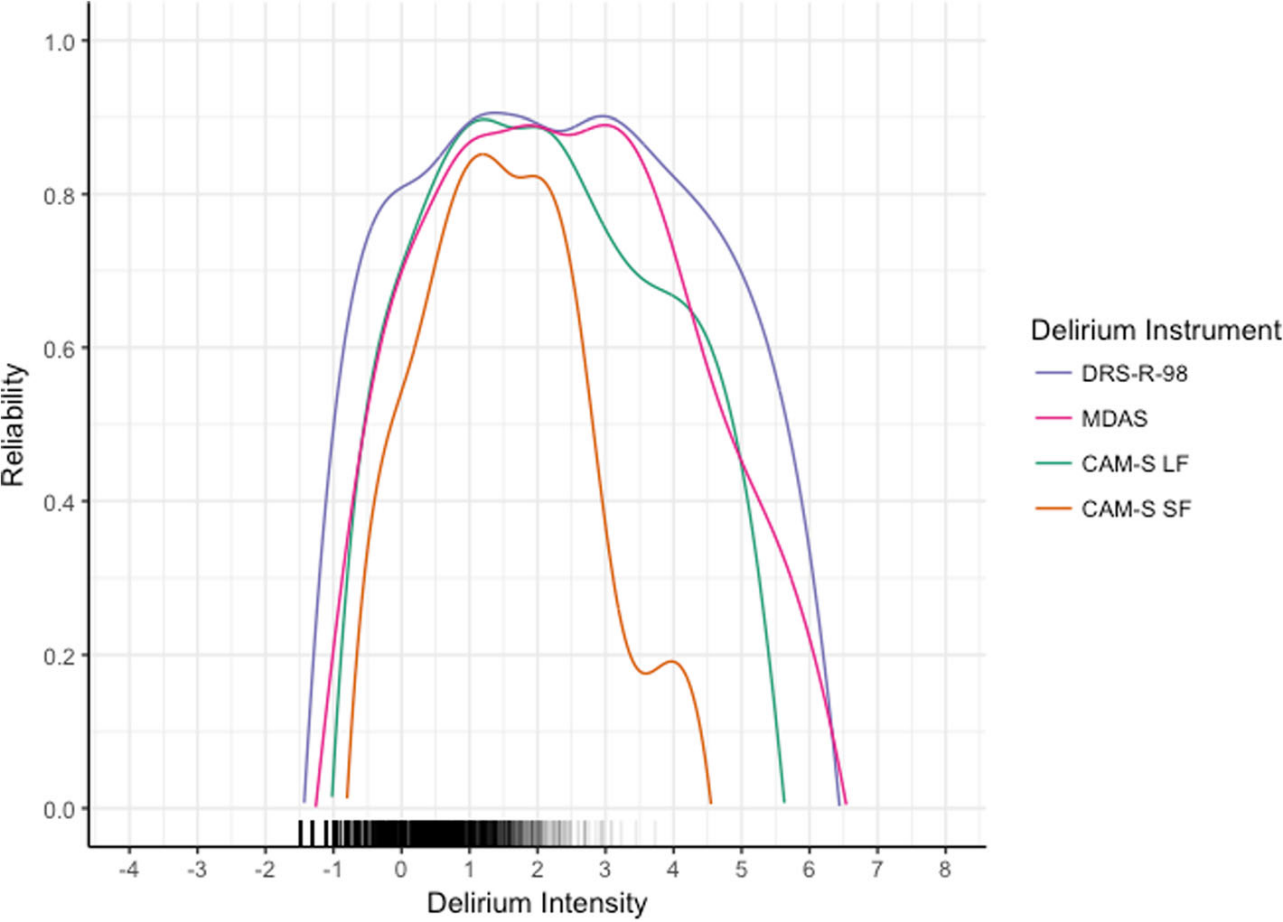
Measurement precision contrasting different delirium intensity instruments: Results from the BASIL study (*N* = 1178 daily observations). Measurement precision or reliability for each delirium instrument is calculated using factor loadings and thresholds from the CFA models that included all rated items. The distribution of delirium intensity scores in the sample is denoted on the bottom of the figure

Figure 3 presents test information functions for the DRS-R-98, MDAS, and CAM-S, calculated using factor loadings and thresholds from the CFA models that included all rated items. This provides information about the measurement precision, or reliability, of the measurement models for each of the instruments. All instruments show more variability towards the edges of the distribution. The DRS-R-98, containing more rated items and more categories for each item than the other instruments, has both the widest breadth of information and the highest information curve. Thus, it has more reliability across a wide breadth of delirium intensity. The information curve for the CAM-S long form provides reliabilities of 70% or higher over approximately 3.5 standard deviation units of the spectrum of delirium intensity. As expected, given less categories and items rated, the CAM-S short form provides less precision than the CAM-S long form but over a comparable range (see Additional file 1: Tables S2-S4). Precision for the MDAS is above 70% across approximately 4 standard deviation units.

**Fig. 4:**
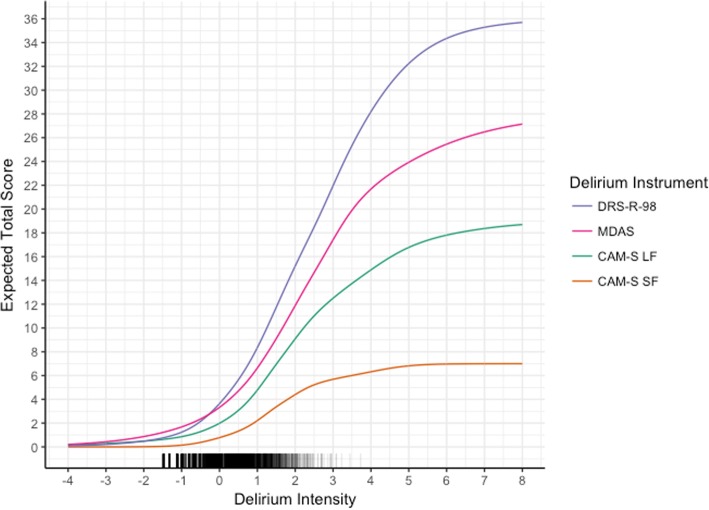
Test characteristic curves for delirium severity instruments: Results from the BASIL study (*N* = 1178 daily observations). These plots for each delirium instrument show the expected score a subject would have on an instrument for a given level of delirium intensity. The distribution of delirium intensity scores in the sample is denoted on the bottom of the figure

Figure 4 shows test characteristic curves for the instruments. These give the expected score a participant would have on an instrument for a given level of delirium intensity. At low levels of delirium intensity, participants endorse few signs/symptoms of delirium, resulting in similar expected scores. As delirium intensity increases, the expected scores diverge. This results from the instruments containing different sets of signs/symptoms of delirium and because of different numbers of response categories. At extremely high levels of delirium intensity, expected scores continue to rise, meaning that at even these very high levels of delirium intensity, participants do not endorse every sign/symptom in each category.

**Fig. 5:**
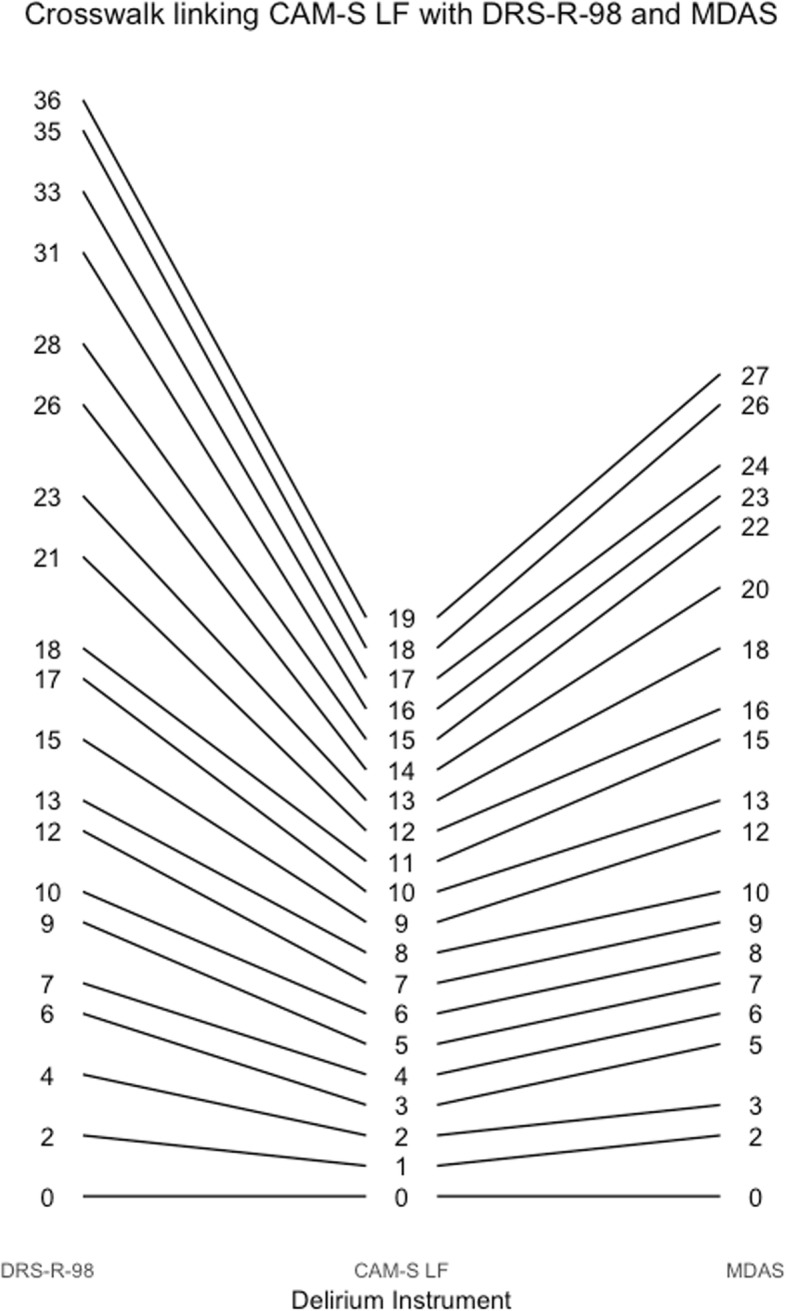
Crosswalk plot linking the CAM-S Long Form with the DRS-R-98 and MDAS: Results from the BASIL study (*N* = 1178 daily observations)

**Fig. 6:**
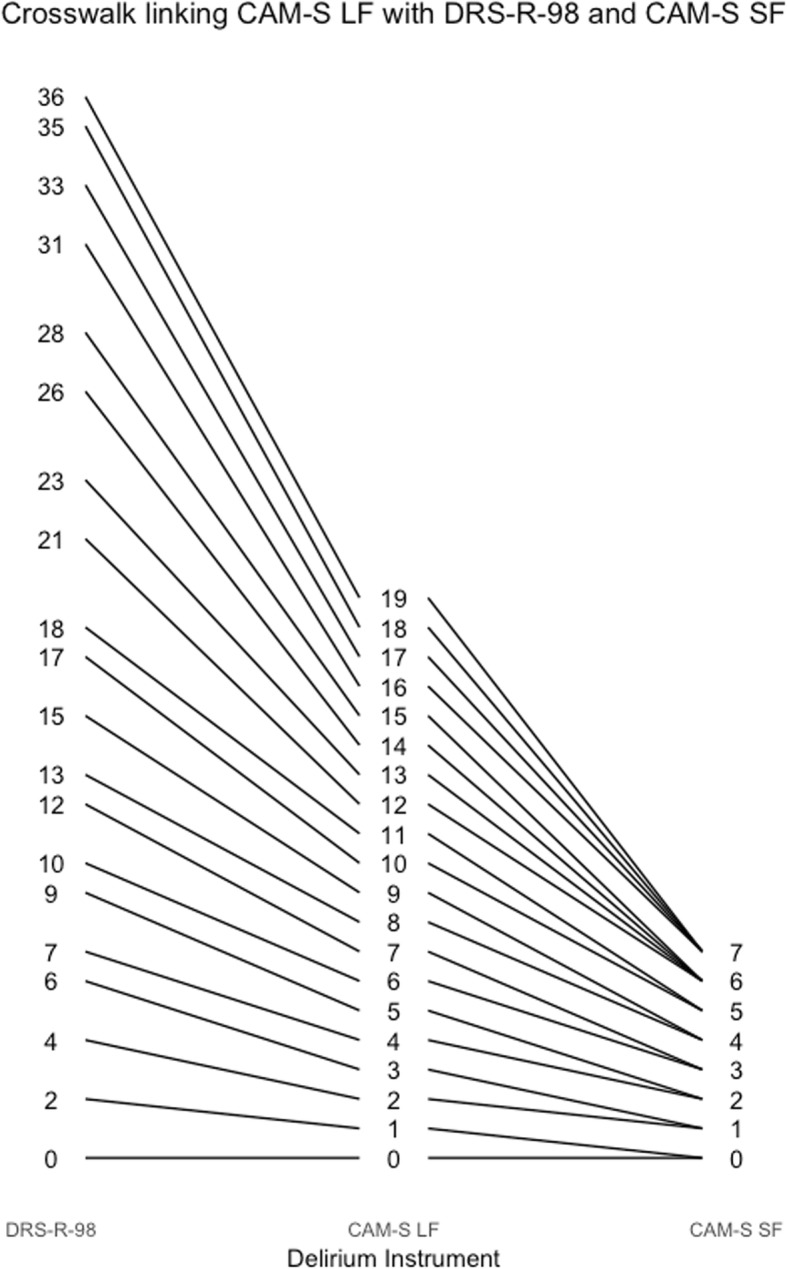
Crosswalk plot linking the CAM-S Long Form with the DRS-R-98 and CAM-S Short Form: Results from the BASIL study (*N* = 1178 daily observations)

**Fig. 7:**
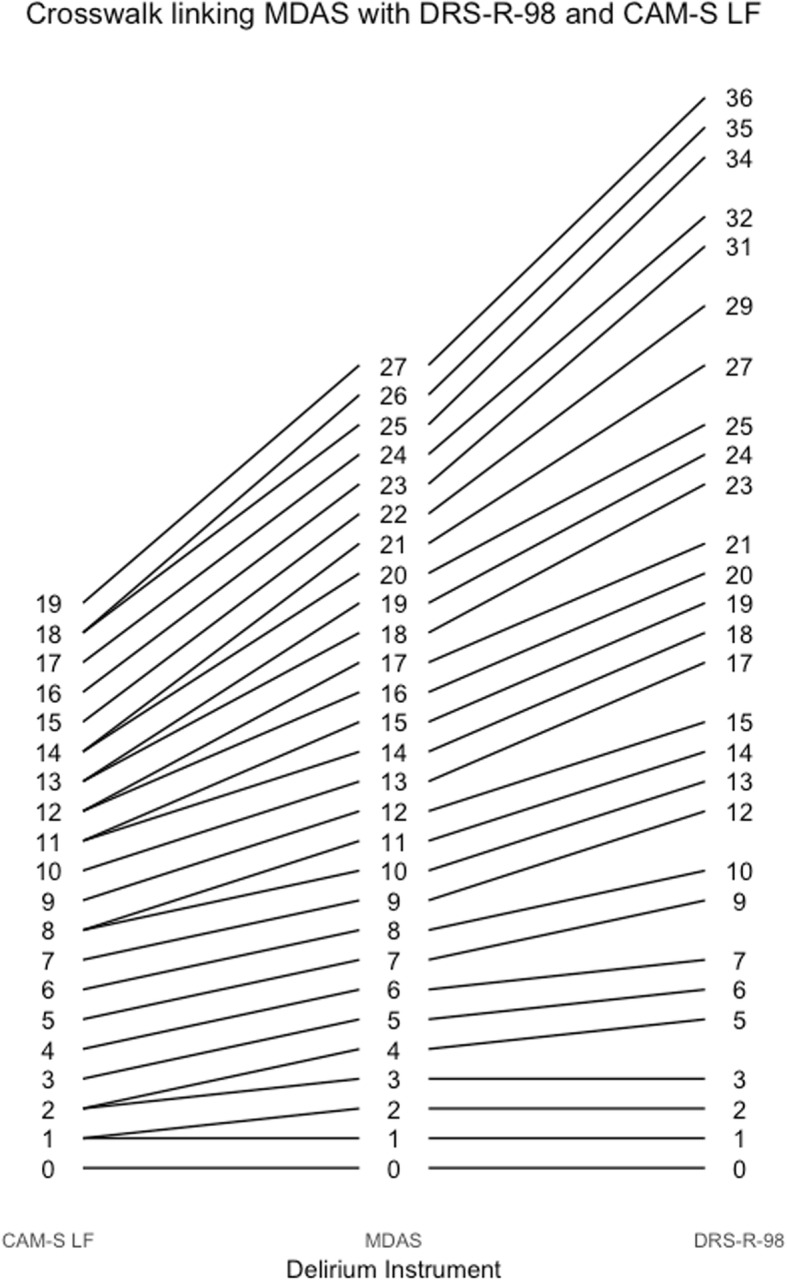
Crosswalk plot linking the MDAS with the CAM-S Long Form and the DRS-R-98: Results from the BASIL study (*N* = 1178 daily observations)

**Fig. 8:**
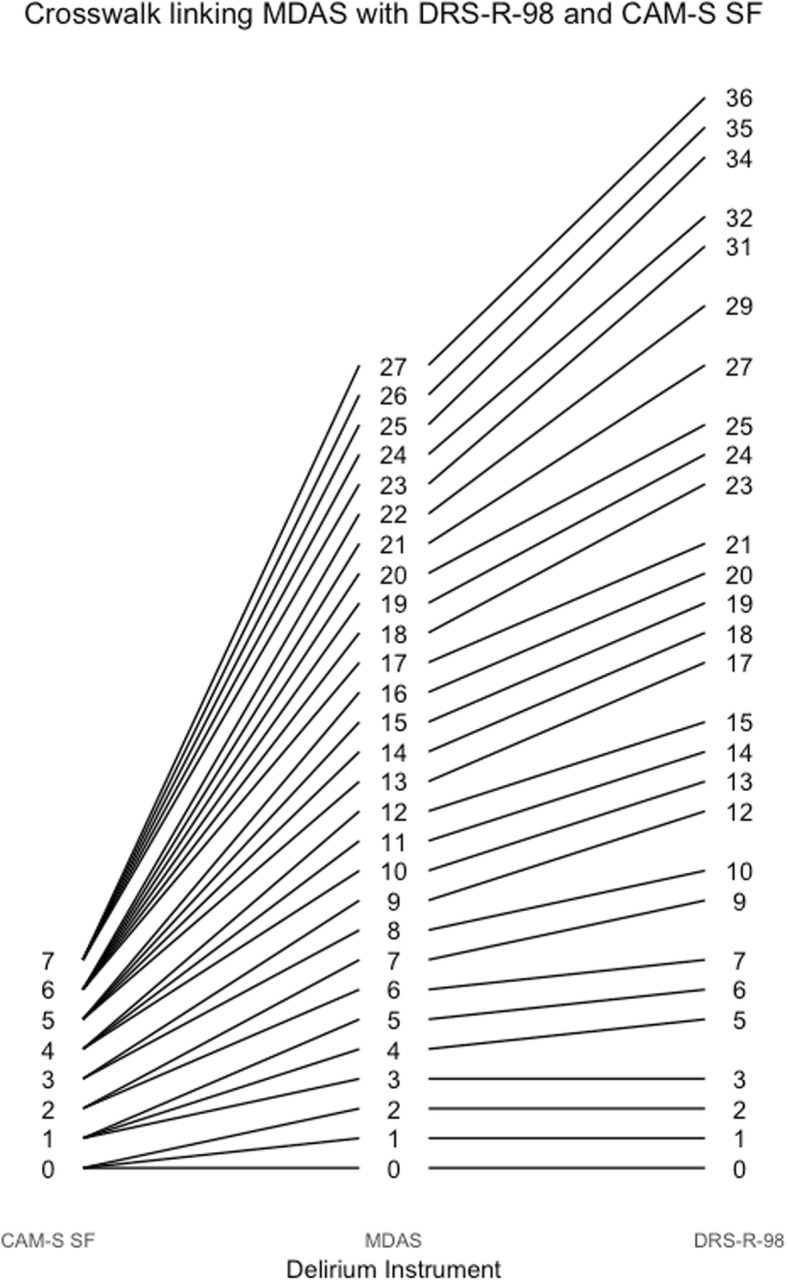
Crosswalk plot linking the MDAS with the DRS-R-98 and the CAM-S Short Form: Results from the BASIL study (*N* = 1178 daily observations)

**Fig. 9:**
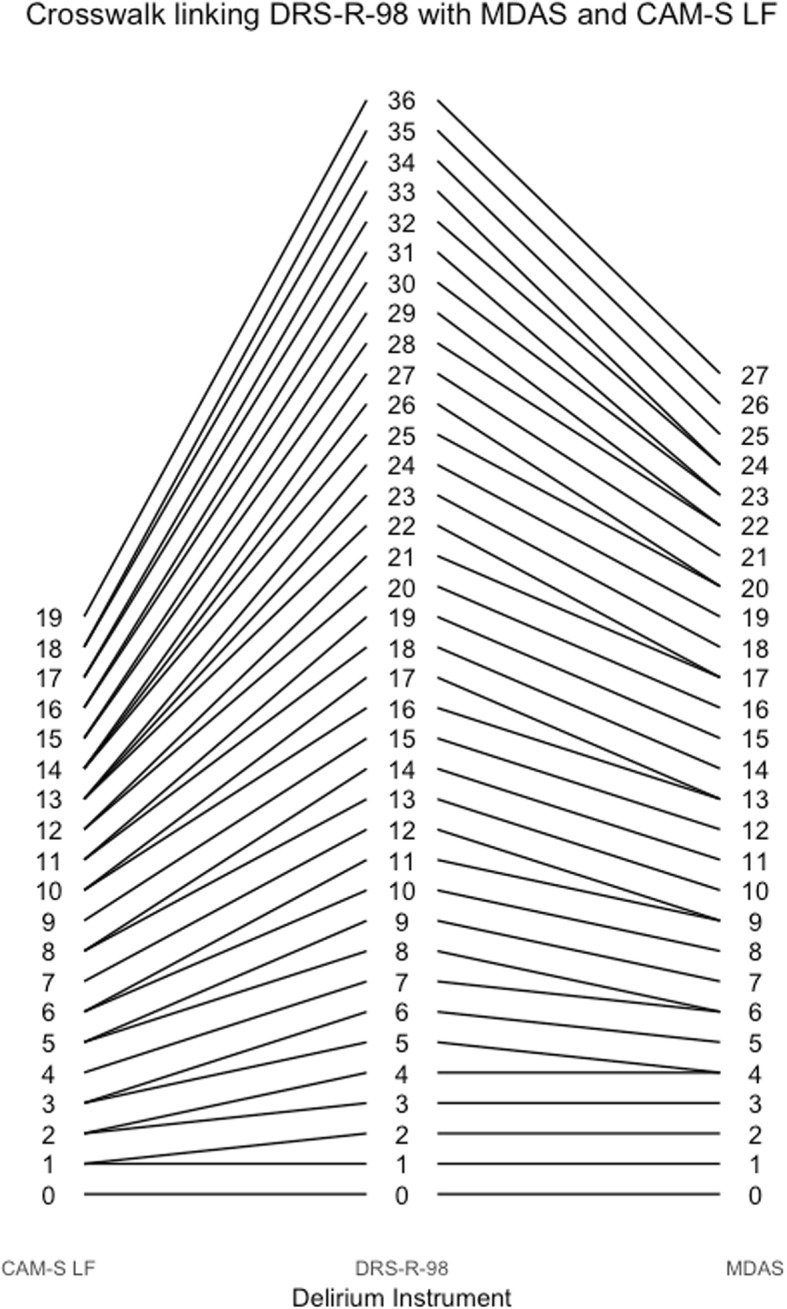
Crosswalk plot linking the DRS-R-98 with the MDAS and the CAM-S Long Form: Results from the BASIL study (*N* = 1178 daily observations)

**Fig. 10:**
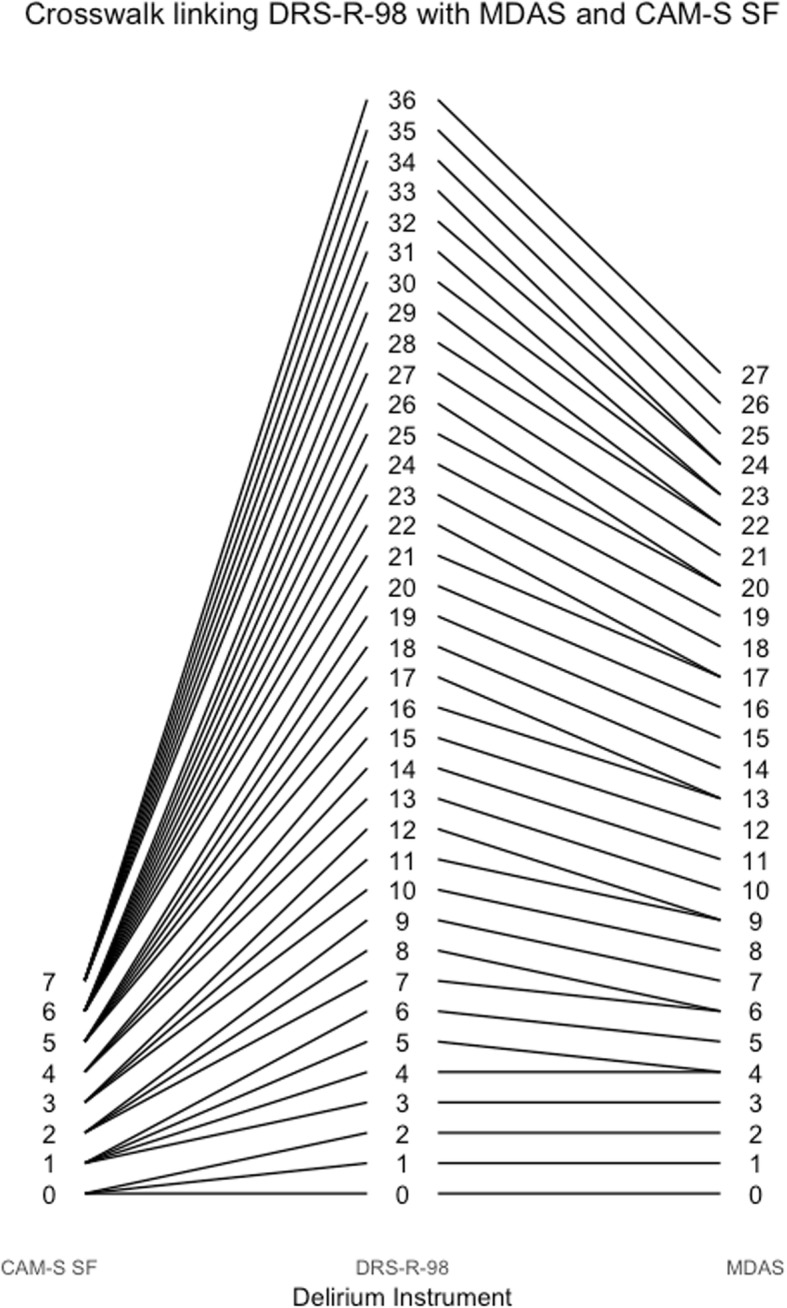
Crosswalk plot linking the DRS-R-98 with the MDAS and the CAM-S Short Form: Results from the BASIL study (*N* = 1178 daily observations)

**Fig. 11:**
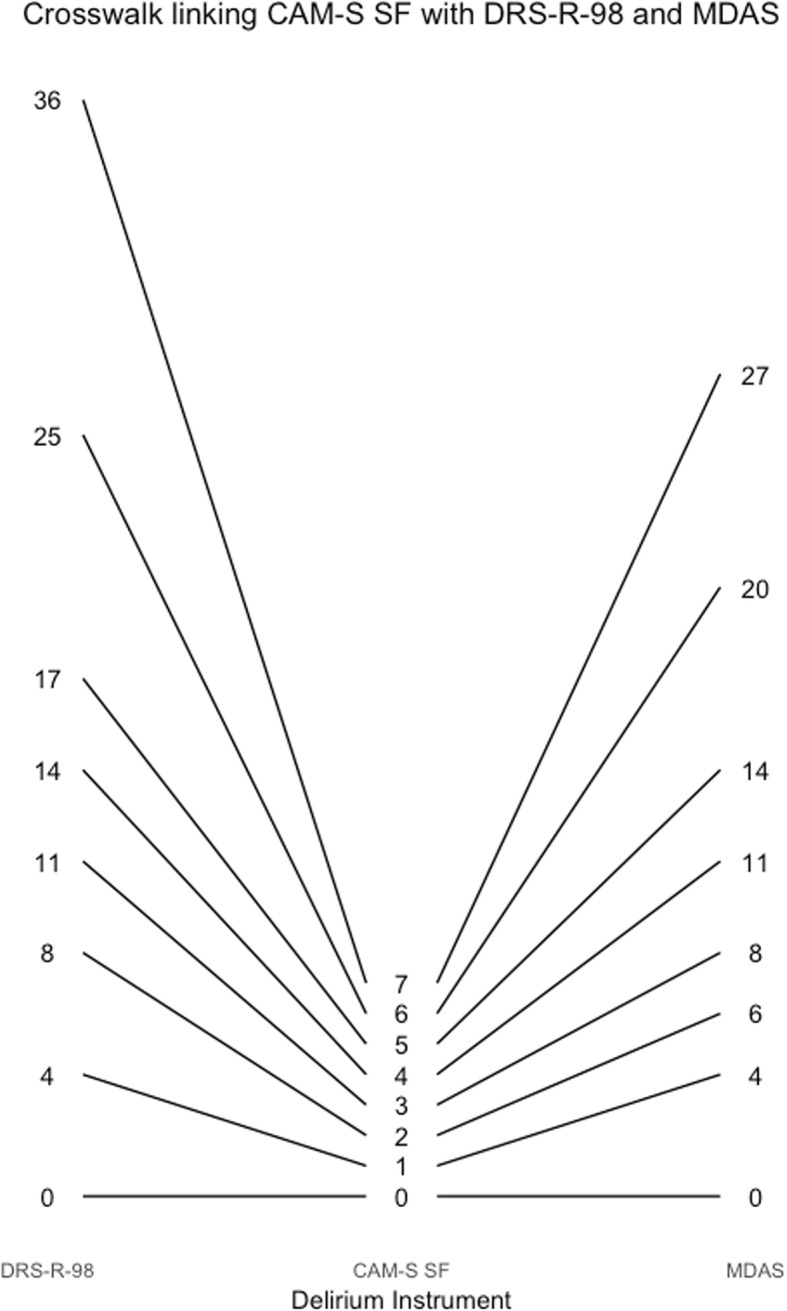
Crosswalk plot linking the CAM-S Short Form with the DRS-R-98 and the MDAS: Results from the BASIL study (*N* = 1178 daily observations)

**Fig. 12:**
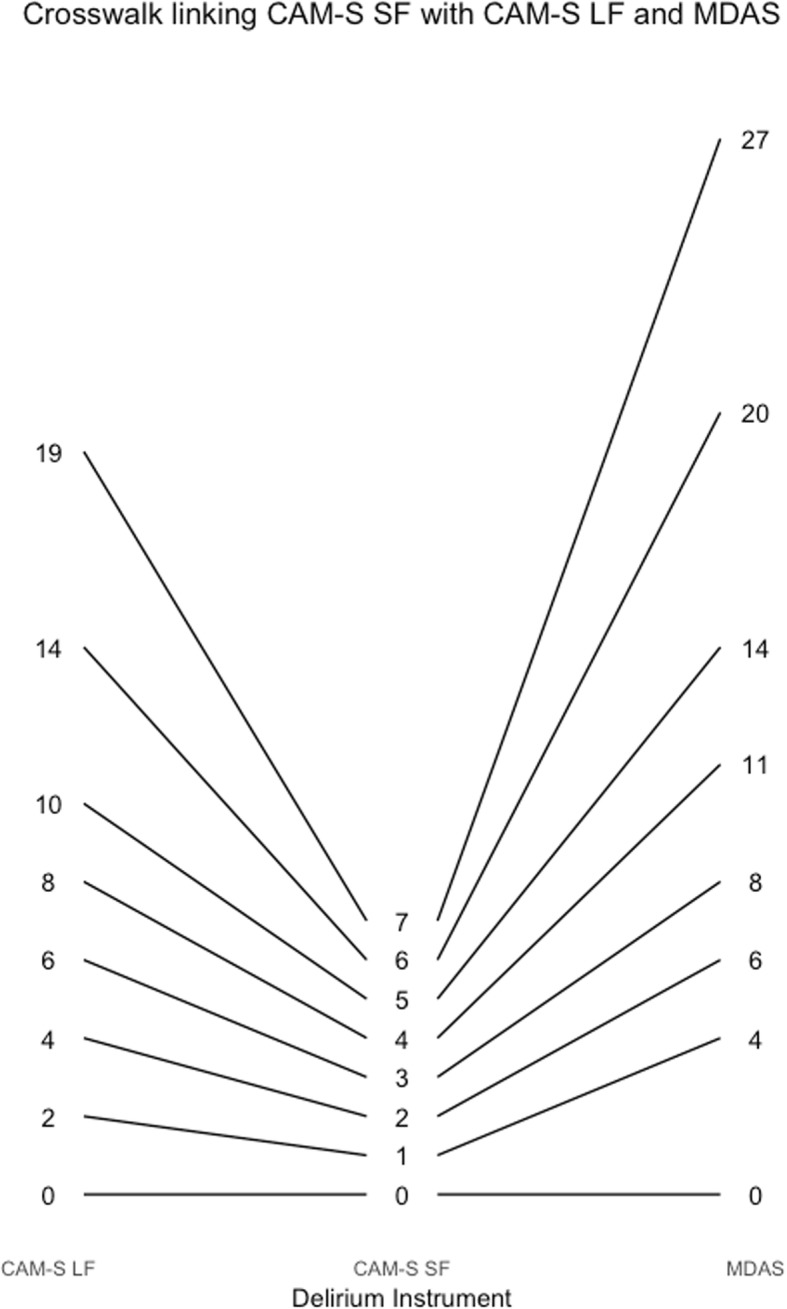
Crosswalk plot linking the CAM-S Short Form with the CAM-S Long Form and the MDAS: Results from the BASIL study (N = 1178 daily observations)

Figure 4 also provides a way to obtain corresponding scores across instruments. For example, a CAM-S long form score of 9 is equivalent to a delirium intensity of 2, which corresponds to a DRS-R-98 score of about 15. Figures 5, 6, 7, 8, 9, 10, 11 and 12 contain more explicit crosswalk plots for each pairwise comparison of scores among the DRS-R-98, MDAS, CAM-S long form, and the CAM-S short form. For example, a CAM-S long form score of 9 corresponds to a DRS-R-98 score of 15 and an MDAS score of 12 (Fig. 5).

**Fig. 13:**
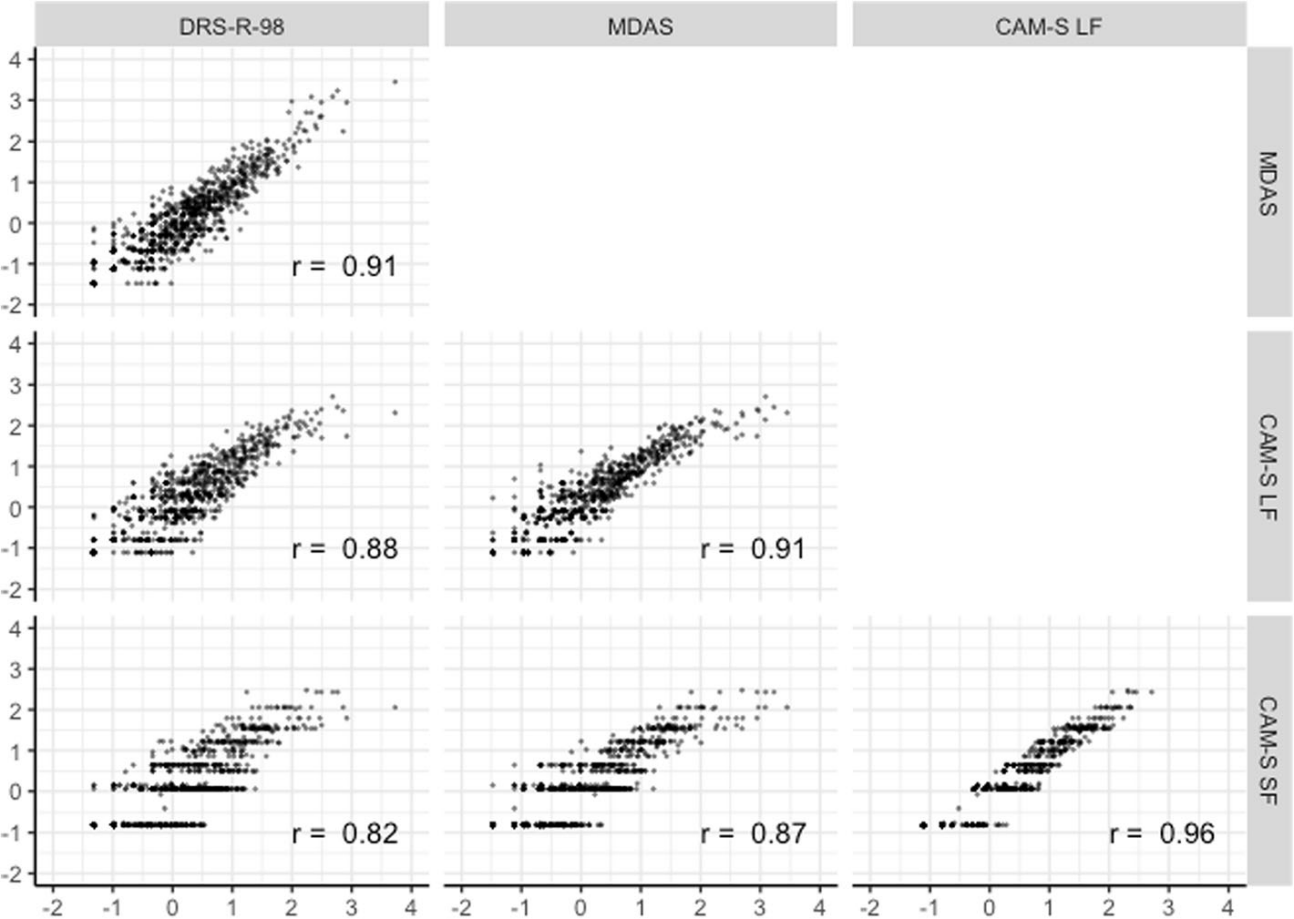
Scatterplot matrix of factor scores from harmonized delirium severity instruments: Results from the BASIL study (N = 1178 daily observations)

Figure 13 shows distributions and correlations among factor scores for delirium intensity estimated from each instrument-specific CFA that used the polytomous outcomes. Correlations were all above 0.82. The CAM-S long form and short form were the most highly correlated (*r* = 0.96).

## Discussion

In this clinical research study of hospitalized older adults, we use modern psychometric methods to map the relative intensity of rated items for three commonly used delirium severity instruments onto a common metric with the goal of comparing or combining studies using different instruments. Relative locations of items help to exemplify the relative intensity of each item, and thus, contributes to the complex and evolving construct of delirium severity. This mapping further enables us to derive crosswalks which allow scores to be easily translated across studies using these diverse delirium severity instruments. All three instruments found less precision toward extremes of the intensity distribution. Having more items with more response categories can improve reliability; thus, the DRS-R-98 has more reliable (stable) ratings across all levels of intensity. The CAM-S is as reliable as the DRS-R-98, but across a narrower range of delirium intensity.

Harmonization of delirium severity measures provides an important advance to the field in several ways. First, it allows comparison of studies using different scales on the same metric; for instance, direct comparison of rates of severe delirium (by the same metric) across studies becomes possible. Second, it facilitates meta-analyses, that is, the ability to combine individual-level data from different studies using different instruments. Another important application is the ability to develop improved measures by choosing optimal items from various measures, such as shorter, faster measures can be developed for quick screening, or longer, more informative, and more accurate measures can be used for reference standard diagnoses.

The goal of this paper was to link three delirium severity instruments, and not to provide recommendations about their relative usefulness. In selecting which delirium severity instrument to use in a specific study, authors will need to decide based on the goals of the study and logistical constraints, such as time for assessment and availability of clinician raters. Importantly, this study does provide valuable information about each instrument, with respect to range of scores, reliability, and intensity of individual rated items, as summarized below.

The DRS-R-98, which has the most questions and response categories, demonstrates the highest information curve along with the broadest distribution of scores, and is uniformly reliable over the widest range of performance of the three instruments. This finding is expected because the DRS-R-98 assesses more symptoms than both the CAM and the MDAS, and rates symptoms with more categories than the CAM. The MDAS [13] had the lowest peak in the information curve, but the second broadest range of test information across the range of delirium severity in our sample. The broader range of measurement of the MDAS compared to the CAM is also expected due to the higher number of response categories. The CAM-S long form had the second highest information curve and provided reliabilities of .70 or higher over approximately 3.5 standard deviation units of the spectrum of delirium intensity. However, the differences in measurement precision we note would likely have minimal impact in clinical practice, with the rare occurrence of such extreme levels of delirium intensity in this sample of hospitalized older adults. Moreover, at such intense levels, the difference in distinguishing a patient with delirium intensity of three versus four standard deviations above the mean would likely have little clinical significance.

The strengths of this study include the detailed data collection on several delirium instruments in a prospective fashion on a diverse and sizeable cohort by rigorously trained, experienced research assistants in a highly consistent manner with high inter-rater reliability. In an innovative approach, we used modern psychometric methods to co-calibrate the different delirium severity scales together using all the available data. Importantly, by allowing factoring loadings in the CFA models to vary from each other, our analyses allow rated items to receive different weights, determined empirically, to measure delirium severity. An advantage of this approach comes by allowing the resulting score to better reflect the underlying construct of delirium severity, as opposed to alternative approaches, such as calculating z-scores of items and averaging them together.

The study has several important limitations. First, not all rated items of delirium intensity had responses in all possible categories; thus, maximum values of the test characteristic curves in Fig. 4 are less than their theoretical maximum values. Second, delirium was assessed using trained lay interviewers and not clinicians. Thus, the DRS-R-98 and MDAS ratings need interpretation as adaptations of the recommended clinician-scored versions. This limitation does not affect the CAM-S ratings, which was designed for either lay interviewers or clinicians. Third, one of our findings in this study is that the cognitive items relate to less intense delirium relative to other rated items. A possible explanation for this finding is that we included patients with dementia in the sample: they likely had more severe cognitive symptoms than patients without dementia, which would lead to lower estimated thresholds in IRT models. Regardless, to give our study real-world applicability and generalizability, our goal was to assess the performance and compare these instruments in a population that included a realistic proportion of persons with baseline mild cognitive impairment and dementia. Fourth, we acknowledge that the 3 delirium instruments were not completely independent, since they were completed by the same raters. Finally, the analysis used only three delirium severity instruments among the many published instruments available. While limited by feasibility constraints, future work will be needed to extrapolate such methods to other important measures.

## Conclusions

In conclusion, this study provides the means to link three widely used measures of delirium severity across a common metric. Linking can facilitate the comparison of results across studies and, ultimately, the combining of studies for integrative analyses and meta-analyses. The ability to combine studies is critical to enhance big data approaches, such as genomic analyses and machine learning, which will be crucial to move the field ahead. While none of the three measures examined were considered ideal from both statistical and logistical perspectives, our results may ultimately help us to improve measurement of delirium severity via future instrument development efforts by recognizing relative difficulties of the various rated items. The development of improved instruments for measuring delirium severity will be essential to provide more nuanced and finely grained outcome measures for clinical trials and pathophysiologic studies which are important to advance the field.

## Additional file

Additional file 1: **Table S1.** Item parameter estimates from instrument-specific confirmatory factor analyses of all items (Analysis step 3). **Table S2.** DRS item frequencies. **Table S3.** MDAS item frequencies. **Table S4.** CAM-S item frequencies. (DOCX 26 kb)

## Abbreviations

ADL: Activities of daily living
BASIL: Better assessment of illness study
BIDMC: Beth Israel deaconess medical center
CAM, CAM-S: Confusion assessment method, confusion assessment method severity
CFA: Confirmatory factor analysis
CFI: Confirmatory fit index
DRS-R-98: Delirium rating scale, revised 1998 version
DSM-III: Diagnostic and statistical manual of mental disorders, third edition
DSM-IV: Diagnostic and statistical manual of mental disorders, fourth edition
IRT: Item response theory
MDAS: Memorial delirium assessment scale
RMSEA: Root mean squared error of approximation
SD: Standard deviation
WLSMV: Weighted least squares, mean and variance adjusted
