# Supplementary material for: Harmonization of delirium severity instruments: a comparison of the DRS-R-98, MDAS, and CAM-S using item response theory
Source: BMC Med Res Methodol. 2018 Sep 10;18:92. doi: 10.1186/s12874-018-0552-4 (PMC6131747; doi:10.1186/s12874-018-0552-4)
Supplement: Supplementary file 1 — Table S1. Item parameter estimates from instrument-specific confirmatory factor analyses of all items (Analysis step 3). Table S2. DRS item frequencies. Table S3. MDAS item frequencies. Table S4. CAM-S item frequencies. (DOCX 26 kb) [file 12874_2018_552_MOESM1_ESM.docx]

Additional file 1

Table S1. Item parameter estimates from instrument-specific confirmatory factor analyses of all items (Analysis step 3)

| Delirium instrument | Item rating | Slope | First threshold | Second threshold | Third threshold |
| --- | --- | --- | --- | --- | --- |
| DRS Model 2 |  |  |  |  |  |
| rdrs01 | Sleep-wake cycle disturbance | 0.93 | -1.31 | 3.26 | 6.57 |
| rdrs02 | Perceptual disturbances and hallucinations | 1.20 | 1.78 | 2.17 | 2.64 |
| rdrs03 | Delusions | 1.51 | 2.66 | 3.33 | 3.68 |
| rdrs04 | Lability of affect | 1.49 | 3.20 | 4.19 | NA |
| rdrs05 | Language | 1.59 | 1.68 | 3.4 | 4.44 |
| rdrs06 | Thought process abnormalities | 3.54 | 1.22 | 1.8 | 2.94 |
| rdrs07 | Motor agitation | 1.63 | 2.88 | 4.06 | NA |
| rdrs08 | Motor retardation | 1.09 | 3.91 | 5.23 | NA |
| rdrs09 | Orientation | 1.75 | 0.96 | 1.8 | 3.98 |
| rdrs10 | Attention | 2.66 | -0.21 | 1.29 | 3.02 |
| rdrs11 | Short-term memory | 1.36 | 0.10 | 1.26 | 1.77 |
| rdrs12 | Long-term memory | 2.44 | -0.13 | 0.63 | 1.08 |
| rdrs13 | Visuospatial ability | 1.61 | 1.71 | 3.1 | 5.16 |
| MDAS Model 2 | |  |  |  |  |
| rmdas01 | Reduced level of consciousness (Awareness) | 2.33 | 2.00 | 2.97 | NA |
| rmdas02 | Disorientation | 1.97 | 0.88 | 1.75 | 2.53 |
| rmdas03 | Short-term memory impairment | 1.34 | -0.07 | 1.02 | 2.59 |
| rmdas04 | Impaired digit span | 1.11 | -1.14 | 1.97 | 5.14 |
| rmdas05 | Reduced ability to maintain and shift attention | 2.37 | 0.16 | 1.41 | 3.14 |
| rmdas06 | Disorganized thinking | 3.50 | 1.08 | 1.89 | 3.07 |
| rmdas07 | Perceptual disturbance | 1.12 | 1.79 | 3.82 | 6.85 |
| rmdas08 | Delusions | 1.48 | 2.62 | 3.61 | 5.44 |
| rmdas09 | Decreased or increased psychomotor activity | 1.32 | 2.71 | 3.93 | NA |
| rmdas10 | Sleep-wake cycle disturbance | 0.74 | -1.71 | 4.12 | 7.99 |

| Table S2, continued | |  |  |  |  |
| --- | --- | --- | --- | --- | --- |
| CAM-S Model 2 | |  |  |  |  |
| rcamlf1a | Acute change | 2.54 | 0.86 | NA |  |
| rcamlf2a | Inattention | 2.39 | -0.21 | 1.41 |  |
| rcamlf3a | Disorganized thinking | 3.82 | 1.18 | 2.06 |  |
| rcamlf4l | Altered level of consciousness (Lethargic) | 1.82 | 2.64 | NA |  |
| rcamlf4v | Altered level of consciousness (Vigilant) | 2.02 | 4.20 | NA |  |
| rcamlf5a | Disorientation | 2.22 | 0.98 | 1.89 |  |
| rcamlf6a | Memory impairment | 1.91 | 0.87 | 2.43 |  |
| rcamlf7a | Perceptual disturbances | 1.33 | 1.85 | 3.88 |  |
| rcamlf8a | Psychomotor agitation | 1.61 | 2.98 | 4.36 |  |
| rcamlf8d | Psychomotor retardation | 0.98 | 4.21 | 6.61 |  |
| rcamlf9a | Sleep-wake cycle disturbance | 0.71 | -1.67 | 4.31 |  |

Table S2: DRS item frequencies

|  |  |  | **Response Frequency** | | | | |
| --- | --- | --- | --- | --- | --- | --- | --- |
| **Item** | **Content** | **Total** | **0** | **1** | **2** | **3** | **Missing** |
| drs01 | Sleep-wake cycle disturbance | 1174 | 302 | 797 | 71 | 4 | 4 |
| drs02 | Perceptual disturbances and hallucinations | 1169 | 991 | 54 | 46 | 78 | 9 |
| drs03 | Delusions | 1169 | 1119 | 30 | 8 | 12 | 9 |
| drs04 | Lability of affect | 1177 | 1150 | 20 | 7 | 0 | 1 |
| drs05 | Language | 1176 | 1024 | 135 | 13 | 4 | 2 |
| drs06 | Thought process abnormalities | 1176 | 1015 | 101 | 55 | 5 | 2 |
| drs07 | Motor agitation | 1176 | 1143 | 27 | 6 | 0 | 2 |
| drs08 | Motor retardation | 1178 | 1150 | 21 | 7 | 0 | 0 |
| drs09 | Orientation | 1172 | 885 | 170 | 112 | 5 | 6 |
| drs10 | Attention | 1177 | 508 | 507 | 153 | 9 | 1 |
| drs11 | Short-term memory | 1170 | 616 | 298 | 89 | 167 | 8 |
| drs12 | Long-term memory | 1158 | 536 | 278 | 135 | 209 | 20 |
| drs13 | Visuospatial ability | 1160 | 1022 | 115 | 22 | 1 | 18 |

Table S3: MDAS item frequencies

|  |  |  | **Response Frequency** | | | | |
| --- | --- | --- | --- | --- | --- | --- | --- |
| **Item** | **Content** | **Total** | **0** | **1** | **2** | **3** | **Missing** |
| mdas01 | Reduced level of consciousness (Awareness) | 1177 | 1114 | 49 | 14 | 0 | 1 |
| mdas02 | Disorientation | 1171 | 900 | 167 | 67 | 37 | 7 |
| mdas03 | Short-term memory impairment | 1170 | 603 | 279 | 221 | 67 | 8 |
| mdas04 | Impaired digit span | 1173 | 344 | 674 | 148 | 7 | 5 |
| mdas05 | Reduced ability to maintain and shift attention | 1178 | 692 | 344 | 131 | 11 | 0 |
| mdas06 | Disorganized thinking | 1178 | 996 | 126 | 49 | 7 | 0 |
| mdas07 | Perceptual disturbance | 1170 | 992 | 151 | 26 | 1 | 8 |
| mdas08 | Delusions | 1170 | 1118 | 38 | 13 | 1 | 8 |
| mdas09 | Decreased or increased psychomotor activity | 1177 | 1118 | 44 | 15 | 0 | 1 |
| mdas10 | Sleep-wake cycle disturbance | 1174 | 300 | 811 | 59 | 4 | 4 |

Table S4: CAM-S item frequencies

|  |  |  | **Response Frequency** | | | |
| --- | --- | --- | --- | --- | --- | --- |
| **Item** | **Content** | **Total** | **0** | **1** | **2** | **Missing** |
| camlf1a | Acute change | 1169 | 874 | 295 | 0 | 9 |
| camlf2a | Inattention | 1176 | 495 | 521 | 160 | 2 |
| camlf3a | Disorganized thinking | 1178 | 995 | 143 | 40 | 0 |
| camlf4l | Altered level of consciousness (Lethargic) | 1178 | 1138 | 40 | 0 | 0 |
| camlf4v | Altered level of consciousness (Vigilant) | 1178 | 1176 | 2 | 0 | 0 |
| camlf5a | Disorientation | 1171 | 900 | 187 | 84 | 7 |
| camlf6a | Memory impairment | 1170 | 855 | 268 | 47 | 8 |
| camlf7a | Perceptual disturbances | 1170 | 1011 | 143 | 16 | 8 |
| camlf8a | Psychomotor agitation | 1178 | 1146 | 28 | 4 | 0 |
| camlf8d | Psychomotor retardation | 1178 | 1148 | 27 | 3 | 0 |
| camlf9a | Sleep-wake cycle disturbance | 1173 | 295 | 811 | 67 | 5 |
